# P07-09 Physical activity prescription for chronic disease in Belgium: results of a 6-month intervention led by physical educators

**DOI:** 10.1093/eurpub/ckac095.109

**Published:** 2022-08-29

**Authors:** Alexandre Mouton, Yoric Petitfrère, Nicolas Franck, Charlotte Ocula, Sara Da, Costa Rocha, Marc Cloes

**Affiliations:** Sport Sciences Departement, University of Liege, Liege, Belgium

**Keywords:** physical activity, prescription, chronic disease, physical educator, physical fitness

## Abstract

**Background:**

Physical activity (PA) prescription has the potential to be an important therapeutic agent for all ages in primary, secondary and tertiary prevention of chronic disease (Thornton et al., 2016). However, physicians report that they do not deliver PA counselling because of limitations in time, knowledge, confidence, and practical tools (Meriwether, Lee, Lafleur, & Wiseman, 2008). Physical educators have the required skills to address those issues in encouraging patients to adopt an active lifestyle that will reduce the incidence of their chronic conditions. In Belgium, physical activity prescription initiatives are emerging: this study aimed at monitoring one of these to ensure the development of evidence-based intervention relying on HEPA best practices.

**Methods:**

With a PA prescription from their doctor, patients with chronic cardiac, neurological, metabolic, oncologic or spinal disease took part to a 6-month intervention supervised by physical educators in a Belgian municipality. The program was composed of weekly sessions of physical exercise focusing on the 4 dimensions of physical fitness according to international recommendations (ACSM, 2016). Monthly motivational sessions were also organized to promote active lifestyle. Monthly assessments included PA level (Ricci & Gagnon,2011), SF-36 (Ware & Sherbourne, 1992), and Senior Fitness Test (Rikli & Jones, 2001).

**Results:**

Preliminary results after one month intervention on 19 patients (68.6 ± 10.8 years) with chronic cardiac (n = 11), neurological (n = 2), metabolic (n = 1), oncological (n = 4) or spinal (n = 1) disease exposed significant improvements of their lower (p = 0.03) and upper (p = 0.01) body strength, and aerobic endurance (p = 0.17). No significant changes were observed for physical activity level and SF-36 scores. Further results will provide information about the impact of motivational sessions on the adoption of an active lifestyle.

**Conclusions:**

Physical activity prescription supervised by physical educators has the potential to bridge the missing link between hospital revalidation performed by health care professionals and autonomous physical activity. If outcomes are favourable, it could provide an advocacy to inspire key decision makers and policies to ameliorate chronic diseases care involving physical educators. Future local interventions should therefore rely on existing guidelines (Inserm, 2019) and support the development of this specific HEPA environment.

